# Whole-Body Vibration Training Versus Kinesiology Taping for Improving Gait Performance in Children With Spastic Cerebral Palsy: A Randomized Controlled Trial

**DOI:** 10.7759/cureus.111030

**Published:** 2026-06-17

**Authors:** Bolarinwa I Akinola, Caleb A Gbiri, Daniel O Odebiyi

**Affiliations:** 1 Physiotherapy, College of Medicine, University of Lagos, Lagos, NGA

**Keywords:** cerebral palsy (cp), gait performance, kinesiology taping, pediatric rehabilitation, whole-body vibration therapy

## Abstract

Background

Gait abnormalities are among the most disabling functional limitations in children with spastic cerebral palsy. Physiotherapeutic interventions that enhance neuromuscular control and biomechanical alignment may improve gait performance. Whole-body vibration training and kinesiology taping have emerged as rehabilitation strategies aimed at improving motor control and functional mobility.

Objective

The objective of this study is to compare the efficacy of whole-body vibration training and kinesiology taping on gait performance in children with spastic cerebral palsy.

Methods

This randomized controlled trial included 86 ambulatory children with spastic cerebral palsy aged 3-12 years classified within Gross Motor Function Classification System levels I-III. Participants were randomly assigned to either a whole-body vibration training group (n = 42) or a kinesiology taping group (n = 44). Interventions were administered three times per week for 12 weeks. Gait performance was assessed using the Edinburgh visual gait score at baseline, six weeks, and 12 weeks. Within-group changes were analyzed using the Friedman test with Dunn-Bonferroni post-hoc comparisons, while between-group differences were evaluated using the Mann-Whitney U test.

Results

Both intervention groups demonstrated improvements in gait performance across the study period. Within-group analysis revealed significant improvements in the Edinburgh visual gait score from baseline to six weeks and baseline to 12 weeks (p < 0.05). However, between-group comparisons showed no statistically significant differences between the whole-body vibration and kinesiology taping groups at baseline, six weeks, or 12 weeks (p > 0.05).

Conclusion

Whole-body vibration training and kinesiology taping both improved gait performance in children with spastic cerebral palsy. However, neither intervention demonstrated clear superiority. These findings suggest that both modalities may serve as effective physiotherapy interventions for improving gait performance in pediatric cerebral palsy rehabilitation.

## Introduction

Cerebral palsy (CP) is the most common cause of motor disability in childhood and is characterized by permanent disorders of movement and posture resulting from non-progressive disturbances in the developing brain [1]. Although the neurological lesion is static, secondary musculoskeletal and neuromuscular impairments frequently develop over time, leading to functional limitations including impaired mobility and gait dysfunction [2].

Spastic cerebral palsy accounts for approximately 70-80% of all CP cases and is commonly associated with neuromuscular impairments such as muscle weakness, impaired selective motor control, abnormal muscle activation patterns, and altered proprioception [3,4]. These impairments contribute to abnormal gait patterns, including equinus gait, crouch gait, stiff-knee gait, and scissoring gait, which significantly limit functional mobility and independence [5].

Gait dysfunction is one of the most disabling consequences of CP and substantially affects participation in daily activities and overall quality of life [6]. Improving gait performance, therefore, represents a primary goal of rehabilitation for children with CP. Conventional physiotherapy interventions such as strengthening exercises, stretching, balance training, and task-specific gait practice remain central to CP management [7].

Whole-body vibration (WBV) training has emerged as a neuromodulatory intervention that stimulates muscle spindles and Ia afferent pathways, facilitating motor neuron excitability through the tonic vibration reflex [8]. Previous studies have reported improvements in muscle strength, balance, and motor performance following WBV therapy in children with cerebral palsy [9].

Kinesiology taping (KT) is another adjunct physiotherapeutic intervention increasingly used in pediatric rehabilitation. KT is believed to enhance proprioceptive feedback, facilitate muscle activation, and support joint alignment through stimulation of cutaneous mechanoreceptors [10]. Studies have suggested that KT may improve posture, trunk stability, and gait symmetry in children with neurological disorders, including CP [11].

Although both WBV and KT have demonstrated positive effects on motor function and gait-related outcomes in children with cerebral palsy, they operate through fundamentally different physiological mechanisms. WBV primarily targets neuromuscular activation through stimulation of muscle spindles and the tonic vibration reflex, whereas KT is proposed to enhance proprioceptive input, optimize muscle recruitment, and improve biomechanical alignment. Given these differences, determining their comparative efficacy is clinically important for evidence-based decision-making, particularly in resource-constrained rehabilitation settings where clinicians may need to prioritize one intervention over another. Furthermore, while previous studies have investigated the individual effects of WBV and KT, direct comparisons of these modalities on gait performance in children with spastic cerebral palsy remain limited. Addressing this gap may provide valuable information regarding the relative benefits of these commonly used adjunct physiotherapy interventions.

Therefore, this study aimed to compare the efficacy of whole-body vibration training and kinesiology taping on gait performance in children with spastic cerebral palsy.

## Materials and methods

Study design

This study employed a parallel-group randomized controlled trial design comparing the effects of whole-body vibration training and kinesiology taping on gait performance. Two groups of research assistants (RAs), a total of six, who are licensed physiotherapists with not less than two years of working experience, were trained on the assessment, treatment procedures, and data collection processes. The first set of RAs served to administer the treatments (four physiotherapists, two per group) while the other set (two physiotherapists) served to assess the participants and were blinded to both the groups and the treatment.

Determination of sample size

The minimum sample size was calculated using the expression of the medium effect.

\[ n = \frac{N(Z_1 + Z_2)^2}{ES^2} \]

Where n = minimum sample size for each group, N = number of groups (2), Z_1_ = α confidence of interval at 0.05 (1.96), Z_2_ = β confidence of interval at 0.20 (0.84); ES = effect size (0.95 using Cohen's standard effect size).

\[ n = \frac{4(1.96 + 0.84)^2}{(0.95)^2} = 34.8 \]

Considering the possibility of 10% attrition rate. The minimum sample size for each group is 34.8+3.48 = 38.28 ~ 39.

Participants

Ambulatory children diagnosed with spastic cerebral palsy were recruited from pediatric rehabilitation centers in Lagos, Nigeria, where they were receiving conventional physiotherapy. The participants included were children aged 3-12 years with a diagnosis of spastic cerebral palsy within the Gross Motor Function Classification System (GMFCS) levels I-III, with the ability to ambulate independently or with assistive devices. Those excluded were children with orthopedic surgery within the previous six months, botulinum toxin injection within the previous six months, or severe cognitive impairment preventing participation.

A total of 86 participants met the inclusion criteria and were randomly assigned using a fish-bowl method into two groups: whole-body vibration group (WBV) (n = 42) and kinesiology taping group (KT) (n = 44). Gait performance was assessed using the Edinburgh visual gait score, a validated observational gait assessment tool used to quantify gait deviations in children with cerebral palsy [12].

**Figure 1:**
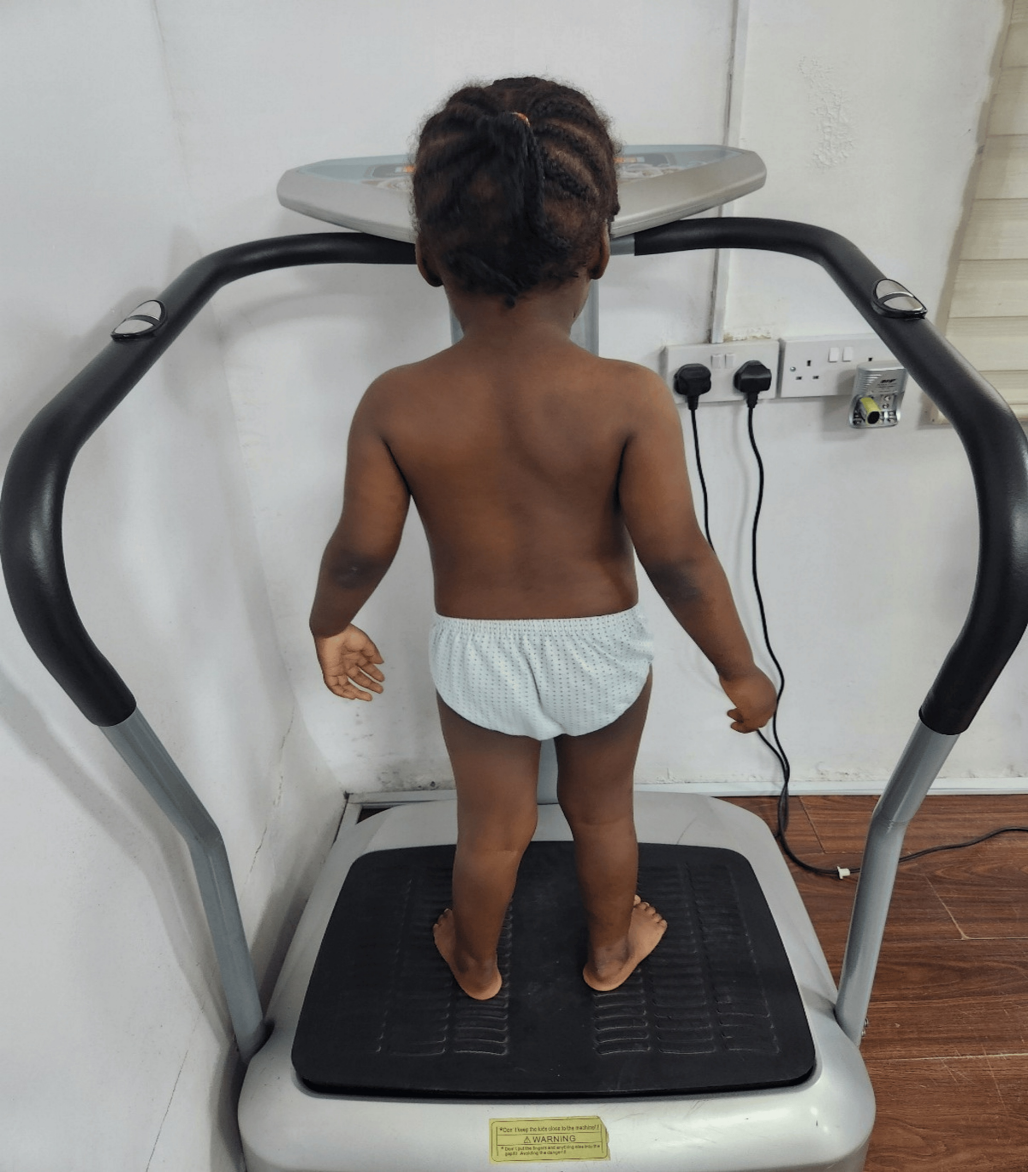
A participant receiving whole-body vibration training

The participants in the whole-body vibration group were made to stand on the WBV machine (model RC-CFM-002, 220-240V, 200 W, 50 Hz) to experience a side-to-side alternating vertical sinusoidal vibration while barefooted (Figure 1). Each participant underwent a test run for familiarization before the study began. The participants were instructed to avoid holding on to the supported rail if possible; however, they were allowed to hold onto the rail if necessary. They were asked to focus on standing with equal weights on both legs as much as possible. Vibration was delivered at a machine preset frequency of 50 Hz with a vertical displacement of 2 mm at a speed of 10m/s (started from 1 and gradually increased to 10) for a duration of 20 minutes according to standard protocol [13]. This was done three times per week for 12 weeks.

**Figure 2:**
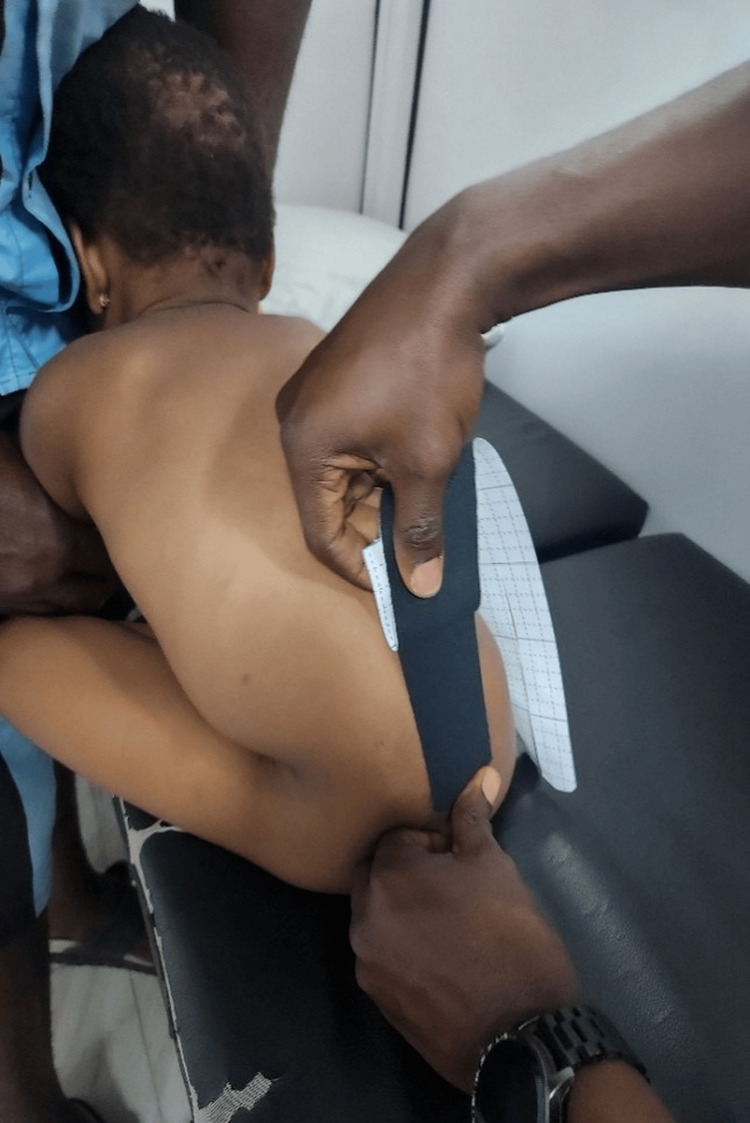
A participant receiving kinesiology taping on erector spinae muscle

Participants in the kinesiology taping (KT) group received taping on their trunk and lower extremities according to Kenzo Kase's Kinesio taping method [14]. Specifically, this was on the erector spinae, gluteus medius, hamstrings, rectus femoris, gastrocnemius, and tibialis anterior. For the erector spinae muscle application, the participants were bent slightly forward in a sitting position, two I-strips tapes were applied 1-2cm lateral to the spinous processes from the sacrum to the 10th or 12th Thoracic vertebrae (T10-T12) using the muscles technique of 30% stretch. For the gluteus medius muscle, two I-strips (one on each side of the spine) were applied from the iliac crest to the greater trochanter. The base of the tape was placed unstretched with the subject in a neutral body position. For the hamstring muscles (medial and lateral), I-strip tape was applied with 10% stretch from the Ischial tuberosity through the posterior thigh and anchored at the fibular head without stretch. For the rectus femoris, the tape was applied from 10 cm below the anterior superior iliac spine to the superior edge of the patella (without tension). The tape was then crossed from the edges of the patella (with maximum tension) and fixed below the inferior edge of the patella while the knee was flexed. For the tibialis anterior muscle, the kinesiology tape was attached along a line that passed the medial epicondyle of the ankle and the medial sole and went to the centerline of the instep with the ankle in a state of plantar flexion. Parents/guardians were instructed to leave the tape on the participants till they come for the next session, when it will be removed and replaced with new ones. They were taught how to maintain them during bathing or play activities. This was done three times per week for a total of 12 weeks (Figure 2).

Statistical analysis

Statistical Package for Social Sciences (SPSS Inc., Armonk, New York, USA) version 25.0 for Windows package program was used to perform data analysis. Descriptive statistics (mean, median, frequency, and percentage) were used to summarise participants' physical characteristics. Within-group comparisons were analyzed using the Friedman test with Dunn-Bonferroni post-hoc comparisons, while between-group comparisons were performed using the Mann-Whitney U test. Statistical significance was set at p < 0.05.

## Results

Results

A total of 86 participants completed the study (WBV = 42 and KT = 44). Baseline demographic and clinical characteristics were comparable between the two groups, as there were no statistically significant differences between groups at baseline in terms of age, height, weight, sex distribution, or Edinburgh visual gait score (EVGS) (p > 0.05) (Table 1).

**Table 1:** Comparison of baseline characteristics of participants in both groups WBV - whole-body vibration; KT - kinesiology taping; EVGS - Edinburgh visual gait score

Variable	WBV (n=42)	KT (n=44)	p-value
Age (years)	7.4 ± 2.6	7.6 ± 2.5	0.73
Height (cm)	118.2 ± 12.4	119.5 ± 11.8	0.68
Weight (kg)	24.8 ± 6.1	25.2 ± 6.3	0.81
EVGS (median)	26.00	28.50	0.72

Within-group comparisons

Within-group analysis using the Friedman test revealed significant improvements in gait performance over time in both intervention groups (Table 2-3).

**Table 2:** Within-group changes in EVGS median scores across intervention duration *=significant at p<0.05 WBV - whole-body vibration; KT - kinesiology taping; EVGS - Edinburgh visual gait score

Group	Baseline	6 Weeks	12 weeks	p-value
WBV	26.00	19.50	13.50	0.001*
KT	28.50	23.00	20.00	0.001*

**Table 3:** Post-hoc test for within-group comparisons of EVGS scores *=significant at p<0.05 WBV - whole-body vibration; KT - kinesiology taping; EVGS - Edinburgh visual gait score

Group	Test variable		Z value	p-value
WBV	Baseline	6-week	-5.65	0.001*
		12-week	-5.51	0.001*
	6-week	12-week	-3.8	0.001*
KT	Baseline	6-week	-5.75	0.001*
		12-week	-5.59	0.001*
	6-week	12-week	-4.63	0.001*

Between-group comparisons

**Figure 3:**
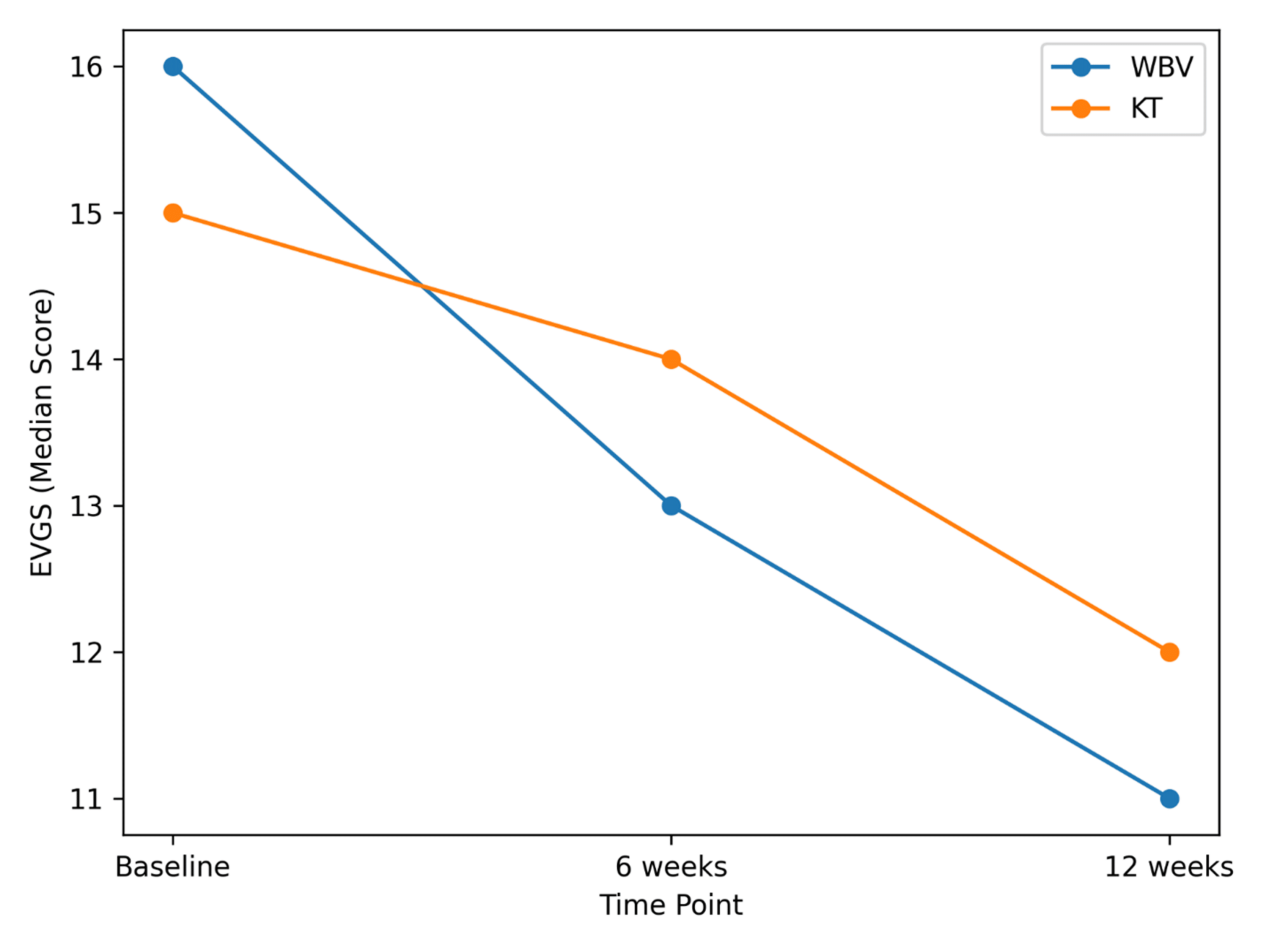
Graphical representation of clinical difference in gait performance between both groups across duration of intervention WBV - whole-body vibration; KT - kinesiology taping; EVGS - Edinburgh visual gait score

Between-group comparisons showed that there was a clinical difference in gait between the two groups (Figure 3). However, no statistically significant difference was found between WBV and KT groups at any assessment time point (Table 4).

**Table 4:** Comparison of EVGS scores between groups WBV - whole-body vibration; KT - kinesiology taping; EVGS - Edinburgh visual gait score

Time point	WBV median	KT median	p-value	
Baseline	26.00	28.50		0.72
6 weeks	19.50	23.00		0.12
12 weeks	13.50	20.00		0.15

## Discussion

The present study compared the efficacy of whole-body vibration training and kinesiology taping on gait performance in children with spastic cerebral palsy. The results demonstrated that both interventions produced significant improvements in gait performance over time. However, no statistically significant differences were observed between the two interventions.

The improvements observed in the WBV group may be explained by the neuromuscular effects of vibration therapy. Whole-body vibration stimulates muscle spindles and Ia afferent pathways, which activate alpha-motor neurons through the tonic vibration reflex. This neuromuscular activation enhances motor unit recruitment, improves muscle strength, and facilitates neuromuscular coordination during functional tasks such as walking [8,9].

Similarly, kinesiology taping demonstrated beneficial effects on gait performance. The mechanism of action of KT is believed to involve stimulation of cutaneous mechanoreceptors, which enhances proprioceptive feedback and improves neuromuscular control. In addition, kinesiology taping may provide mechanical guidance and alignment support to muscles and joints during movement, thereby improving gait stability and efficiency [10,11].

Recent evidence supports the beneficial effects of whole-body vibration (WBV) on functional outcomes in children with cerebral palsy. In a network meta-analysis, Han et al. [8] reported that systemic vibration was among the most effective interventions for improving balance and gait in individuals with cerebral palsy, suggesting that vibration-induced neuromuscular activation may positively influence functional mobility. Similarly, Pulay et al. [9] demonstrated through a systematic review and meta-analysis that WBV can improve mobility, balance, and gross motor function, although considerable heterogeneity existed across intervention protocols and outcome measures. More recently, Huang et al. [15]. found that WBV improved walking speed, Timed Up and Go performance, postural control, and gross motor function in children with cerebral palsy, although the certainty of evidence ranged from very low to low. These findings support the significant within-group improvements observed in the WBV group in the present study and reinforce the role of WBV as a useful tool in pediatric neurorehabilitation. However, the variability in reported treatment effects across studies may explain why WBV did not demonstrate superiority over kinesiology taping in the current trial.

Evidence regarding kinesiology taping (KT) has also strengthened in recent years. A systematic review by Agyenkwa et al. [10] concluded that KT may improve balance, gait, and gross motor function in the lower limbs of children with cerebral palsy, particularly when applied repeatedly as an adjunct to conventional physiotherapy. Likewise, Lin et al. [11], in a recent systematic review and meta-analysis of randomized controlled trials, reported significant improvements in gross motor function, balance ability, and gait-related outcomes following KT intervention. These findings are consistent with the significant improvements observed in the KT group in the present study and support the proposed mechanisms of enhanced proprioceptive feedback, sensory stimulation, and biomechanical alignment.

Despite the positive findings associated with both interventions, the absence of statistically significant between-group differences in the present study suggests that WBV and KT may produce comparable functional benefits through different physiological pathways. While WBV primarily acts through neuromuscular excitation and enhanced motor unit recruitment, KT appears to exert its effects through improved proprioception, postural control, and movement guidance. Therefore, the comparable improvements observed in gait performance may reflect convergence of these distinct mechanisms toward a common functional outcome. This interpretation is supported by the contemporary literature, which consistently demonstrates the benefits of both interventions but does not provide strong evidence that one modality is clearly superior to the other for improving gait performance in children with cerebral palsy.

Limitations

This study did not include a non-intervention control group, limiting the ability to determine the extent to which the observed improvements were attributable solely to the interventions rather than natural developmental changes or other external influences. Also, gait performance was assessed using the Edinburgh visual gait score, an observational clinical measure. Although the EVGS is a valid and reliable tool for gait assessment in children with cerebral palsy, instrumented three-dimensional gait analysis would have provided more detailed biomechanical information regarding treatment-related changes. Finally, the follow-up period was relatively short, and therefore, the long-term sustainability of treatment effects remains unknown. Future studies should incorporate longer follow-up periods and instrumented gait analysis.

## Conclusions

Whole-body vibration training and kinesiology taping both significantly improved gait performance in children with spastic cerebral palsy. However, neither intervention demonstrated superiority over the other. These findings suggest that both modalities may serve as effective physiotherapy interventions for improving gait performance in pediatric cerebral palsy rehabilitation.
